# Inhibition of HTLV-1 Infection by HIV-1 First- and Second-Generation Integrase Strand Transfer Inhibitors

**DOI:** 10.3389/fmicb.2019.01877

**Published:** 2019-08-13

**Authors:** Michał S. Barski, Jordan J. Minnell, Goedele N. Maertens

**Affiliations:** Division of Infectious Diseases, Section of Molecular Virology, Department of Medicine, St Mary’s Hospital, Imperial College London, London, United Kingdom

**Keywords:** HTLV-1, integrase, INSTI, raltegravir, elvitegravir, bictegravir, PVL

## Abstract

More than 10 million people worldwide are infected with the retrovirus human T-cell lymphotropic virus type 1 (HTLV-1). Infection phenotypes can range from asymptomatic to severe adult T-cell leukemia/lymphoma (ATLL) and HTLV-1-associated myelopathy. HTLV-1, like human immunodeficiency virus type 1 (HIV-1), is a blood-borne pathogen and viral infection happens in a similar fashion, with the major mode of transmission through breastfeeding. There is a strong correlation between time of infection and disease development, with a higher incidence of ATLL in patients infected during childhood. There is no successful therapeutic or preventative regimen for HTLV-1. It is therefore essential to develop therapies to inhibit transmission or block the onset/development of HTLV-1 associated diseases. Recently, we have seen the overwhelming success of integrase strand transfer inhibitors (INSTIs) in the treatment of HIV-1. Previously, raltegravir was shown to inhibit HTLV-1 infection. Here, we tested FDA-approved and two Phase II HIV-1 INSTIs *in vitro* and in a cell-to-cell infection model and show that they are highly active in blocking HTLV-1 infection, with bictegravir (EC_50_ = 0.30 ± 0.17 nM) performing best overall. INSTIs, in particular bictegravir, are more potent in blocking HTLV-1 transmission than tenofovir disproxil fumarate (TDF), an RT inhibitor. Our data suggest that HIV-1 INSTIs could present a good clinical strategy in HTLV-1 management and justifies the inclusion of INSTIs in clinical trials.

## Introduction

Human T-cell lymphotropic virus type 1 (HTLV-1) belongs to the delta-retrovirus genus and is the second most clinically relevant retrovirus after human immunodeficiency virus type 1 (HIV-1). Like HIV-1, HTLV-1 is a blood-borne pathogen and is transmitted horizontally by sexual intercourse, contact with infected blood, and vertically from mother to child during breastfeeding and labor. HTLV-1 is a highly potent oncogenic virus, despite the fact that pathology develops only in about 10% of carriers. The two main pathologies associated with infection are an aggressive form of blood cancer – adult T-cell leukaemia/lymphoma (ATLL) (Mahieux and Gessain, 2007) and a spectrum of neuromuscular disorders called HTLV-associated myelopathy/tropical spastic paraparesis (HAM/TSP) (Matsuura et al., 2016). There is no therapeutic or preventative treatment for HTLV-1 and disease prognosis is very poor – particularly for acute ATLL, where the median survival rate is about 2 months (Beltran et al., 2011).

Human T-cell lymphotropic virus type 1 infection is of significant epidemiological importance. It is estimated that between 10 and 20 million people are infected worldwide (Gessain and Cassar, 2012). This figure is most certainly underestimated, since infection can be asymptomatic and diagnosis and lack of understanding and care in public health (Zihlmann et al., 2012) in many countries is still poor. In some endemic areas, the prevalence of HTLV-1 infection is staggeringly high [above 35% in regions of central Australia (Einsiedel et al., 2014, 2018) and above 5% in the Kyushu island of Japan (Gessain and Cassar, 2012)]. A recent open letter, instigated by the co-discoverer of the retrovirus family – Robert Gallo (Martin et al., 2018), appeals to the scientific and medical community to take urgent measures in order to prevent the further spread of HTLV-1.

Central to the life cycle of HTLV-1 is the integrase (IN) enzyme – a nucleotidyl transferase responsible for insertion of the DNA copy of the viral RNA genome into host cell DNA. Following reverse transcription, IN engages with the viral long terminal repeat (LTR) ends of the DNA thereby forming a synaptic complex also called the intasome. The integration reaction takes place in two steps: (i) in the 3′ processing reaction, IN removes two (or, depending on the genus, three) nucleotides following the invariant CA-dinucleotide thereby generating 3′-OH groups necessary for the next step in the integration reaction; (ii) following nuclear entry, the intasome engages with host chromatin and the reactive 3′-OH groups attack the scissile phosphodiester backbone on opposing strands of the host DNA stably inserting the vDNA into the host genome (Li et al., 2006; Maertens et al., 2010; Cherepanov et al., 2011). The retroviral IN is composed of three domains, with the central catalytic core domain (CCD) being the most conserved. The CCD encompasses the integrase catalytic triad (DDE motif), which coordinates two catalytically critical divalent metal ions (usually Mg^2+^). The irreversible insertion of both vDNA ends into host DNA, termed “concerted integration,” results in a stably integrated provirus which is copied with every cell division. The proviral expression products further stimulate cell proliferation and lead to the smoldering, mitotic spread of HTLV-1 (Azran et al., 2004; Bindhu et al., 2004). Due to the imperative role of IN in successful retroviral infection, several IN strand transfer inhibitors (INSTIs) have been developed and released for use in HIV-1 therapy, and have since revolutionized the treatment of this virus (Summa et al., 2008; Serrao et al., 2009; Gillette et al., 2014). These diketo acid derivatives utilize the active site pocket in the intasome through simultaneous chelation of the catalytic pair of Mg^2+^ ions by the diketo group oxygens, and π-π stacking of their halobenzyl moiety to the terminal vDNA adenine. INSTIs compete for binding with the target host DNA and displace the reactive 3′ termini of vDNA away from the active site (Hare et al., 2010a,b). Successive iterations of INSTIs developed have addressed the issues of drug resistance, safety and dissociation rate in HIV-1 treatment.

In Rabaaoui et al. (2008), first reported on the inhibition of HTLV-1 integration by styrylquinolines and diketo acids, the latter being precursors of the currently used HIV-1 INSTIs. More recently, raltegravir and MK-2048 were shown to efficiently block HTLV-1 infection (Seegulam and Ratner, 2011). Here, we present data on a comprehensive panel of INSTIs that are FDA-approved or awaiting approval for treatment of HIV-1 infection that were tested for their efficacy to block HTLV-1 integration *in vitro* and in cell-to-cell infection. We describe an optimized *in vitro* assay that allows for easy screening of INSTI efficacy and IC_50_ determination. *Ex vivo* experiments in Jurkat cells infected with HTLV-1 by cell-to-cell transmission indicate that the INSTI inhibition profiles are very similar to those of HIV-1. Taken together, we present data that support the use of INSTIs as prophylactic treatment, and may justify their use in clinical trials.

## Materials and Methods

### Expression and Purification of Recombinant HTLV-1 Integrase

Human T-cell lymphotropic virus type 1 IN (GenBank ID: AHX00119.1) was expressed and purified from pET28a-SUMO-HTLV-1 IN as described previously (Maertens, 2016) with minor modifications. Briefly, following elution from the cation exchange column, positive fractions containing HTLV-1 IN were pooled and dialyzed against 25 mM piperazine-N,N′-bis(2-ethanesulfonic acid) (PIPES) pH 6, 300 mM NaCl at 4°C overnight. The resulting solution was injected onto an S200 16/60 Superdex size-exclusion column (GE Healthcare, United Kingdom) pre-equilibrated in dialysis buffer. The peak fractions corresponding to IN were pooled and concentrated in a 10 kDa molecular weight cut-off centrifugal ultrafiltration device to a concentration of about 10 mg/mL and supplemented with 2 mM DTT and 10% glycerol. Protein was aliquoted, flash frozen in liquid nitrogen and stored at −80°C until further use. The B′γ regulator subunit of PP2A (further referred to as B′γ) was purified as described previously (Maertens, 2016).

### Integrase Strand Transfer Assays

Donor DNA mimicking the U5 3′-processed LTR ends of the viral DNA copy was prepared by annealing oligonucleotides (Supplementary Table S1), in 100 mM Tris pH 7.4, 400 mM NaCl. The optimized conditions for the strand transfer reactions are as follows: a solution was prepared containing 73 mM PIPES pH 6.0, 175 mM NaCl, 16.7 mM MgCl_2_, 5.8 μM ZnCl_2_, 12.8 mM DTT, 0.53 μM donor DNA, and 0.8 μM HTLV-1 integrase. Following a 30 min room temperature incubation in the presence or absence of INSTIs in the final concentration of 5% DMSO, the reaction was initiated by addition of 300 ng of pGEM-9Zf(−) supercoiled plasmid DNA. The total volume of each reaction was 150 μL. Reactions were carried out at 37°C for 30 min and were processed as described previously (Maertens et al., 2010). All reactions were performed in these conditions unless otherwise stated in the Figure legend. Reactions that included B′γ were done with equimolar ratio of IN to B′γ. The precipitated DNA was resuspended in agarose loading dye and analyzed on a 1.5% agarose gel run in 1× TAE buffer and stained with ethidium bromide. Bands corresponding to products of concerted integration were quantified by densitometry in ImageLab 4.1 (Bio-Rad). All experiments were done in triplicate.

Quantified data points were fitted to a dose-response curve in Prism 7. Cumulative standard deviation for each drug was calculated as an average of the upper limit and lower limit values:

$$\text{Lower limit}=\frac{E\,C_{50}}{10^{\log S\,E\,\left(E\,C_{50}\right)}}$$

$$\text{Upper limit}=E\,C_{50}\times 10^{\log S\,E\,\left(E\,C_{50}\right)}$$

Where SE denotes the standard error computed by Prism.

### Cell Lines and Media

The MT-2 and Jurkat (E6.1) T cell lines (ATCC) were maintained in RPMI supplemented with 10% foetal bovine serum (FBS), 100 U penicillin 100 μg/mL streptomycin and 0.25 μg/mL fungizone. Cells were cultured at 37°C with 5% CO_2_ in a humidified atmosphere.

### HTLV-1 Infection by Cell-to-Cell Transmission

Jurkat cells were pre-treated with increasing amounts of INSTI (0.2 pM–2 μM in DMSO), or the carrier (DMSO) for 24 h. MT-2 cells were resuspended to a concentration of 2 × 10^6^ cells/mL and exposed to a sub-lethal dose of gamma-irradiation (40,000 Rad). Cells were resuspended in serum-free RPMI to a concentration of 2 × 10^6^ cells/mL in the presence of drug, and 0.5 × 10^6^ of each cell type were co-cultured for 18 h. Following co-culture, cells were washed in PBS and resuspended in 1 mL of depletion buffer (0.1% FBS, 2 mM EGTA, PBS) before gentle tumbling (1 h, 4°C) with 25 μL of anti-CD25 + magnetic beads (DynaBeads, Thermo Fisher Scientific) to deplete MT-2 cells. Following depletion, the unbound fraction, i.e., Jurkat cells were maintained in RPMI with the drug present for 16 days before genomic DNA harvest (DNeasy kit, Qiagen). All experiments were done in triplicate. Depletion of MT-2 cells was verified by FACS. For cellular toxicity measurements, Jurkat cells were grown in the presence of the INSTIs as described above. Viability was measured by live dead stain (Hoechst 33258) and analyzed by FACS.

### FACS Protocol for MT-2 and Jurkat Identification

Samples of Jurkat and MT-2 cultures pre- and post-depletion were immunostained for CD3 and CD25 to determine the efficiency of anti-CD25 magnetic depletion. 1 × 10^6^ cells were washed extensively in PBS + 3% FBS before incubation with 0.05 μg Alexa 647-conjugated anti-CD3 + (Biolegend, clone UCHT1) and 0.1 μg Alexa488 conjugated anti-CD25 + (Biolegend, clone BC96) antibodies in the dark for 30 min. Isotype control antibodies were used to determine specificity of the antibodies and staining protocol. Following extensive washing, samples were fixed in 2% formaldehyde for FACS analysis. Single cells were gated, CD3 and CD25 were identified by emission/excitation at 640/670 nm and 488/530 nm, respectively. Data were analyzed on FlowJo software version 10.7.

### Determination of HTLV-1 Proviral Load and Alu-PCR

Proviral load (PVL) was estimated as previously described (Manivannan et al., 2016; Rowan et al., 2016). Genomic DNA concentrations were measured by nanospectroscopy (DeNovix), and samples were diluted to 5 ng/μL for real-time PCR (qPCR) analysis. qPCR reactions were performed for both the HTLV-1 *tax* gene and the human *GAPDH* gene (Supplementary Table S2). Gene copy numbers were determined by comparison to standard curves generated from a clone with a known single integration site (clone 11.50, courtesy of Prof. Charles Bangham). PVL was calculated by comparison of *tax* and *GAPDH* copy numbers as a fraction, multiplied by 100 to generate a percentile value assuming a single copy of *tax* and two copies of *GAPDH* per infected cell (Lairmore et al., 1989). Alu-PCR was performed as described previously (Alais et al., 2015), with the modification that the nested PCR was also run as a qPCR using SYBR-green (primers used are shown in Supplementary Table S2). Integrated provirus copy numbers were normalized to *GAPDH*, and DMSO treated HTLV-1 infected Jurkat cells were arbitrarily set to 100%. Averages and standard deviations of three independent experiments are shown. *P*-values were calculated using the student’s *t*-test.

### Construction of Integrase Structural Models

Human T-cell lymphotropic virus type 1 amino acid sequence (residues 53–216, corresponding to the catalytic domain) was submitted for automatic homology model building to the on-line software Phyre2 (Kelley et al., 2015). The search yielded a high-quality model with confidence score of 100% and 99% coverage. For structural comparisons, a previously reported structural model of HIV-1 intasome was used, which was constructed on the basis of the PFV intasome crystal structure in complex with raltegravir and elvitegravir (Krishnan et al., 2010). HTLV-1 and HIV-1 models were aligned and annotated in PyMol (Schrodinger, 2015). Multiple sequence alignments were conducted in Jalview (Waterhouse et al., 2009) with the MAFFT algorithm (Katoh and Standley, 2013). ESPript was used for graphical representation of the alignments (Gouet et al., 1999). The sequence conservation scores annotation on the structural models was conducted with the Alebrijes script^1^.

## Results

### *In vitro* Catalytic Strand Transfer Activity of HTLV-1 IN

Full-length wild type HTLV-1 IN was recombinantly expressed, purified, and characterized. HTLV-1 IN *in vitro* activity on radiolabeled vDNA donor mimics (Balakrishnan and Jonsson, 1997; Muller and Krausslich, 1999), as well as IN strand transfer activity dependence on the human host factor B′γ have been reported previously (Maertens, 2016). Here, we have attempted to further characterize the strand transfer activity of HTLV-1 IN and optimized the assay for efficient testing of integrase inhibitors.

**FIGURE 1:**
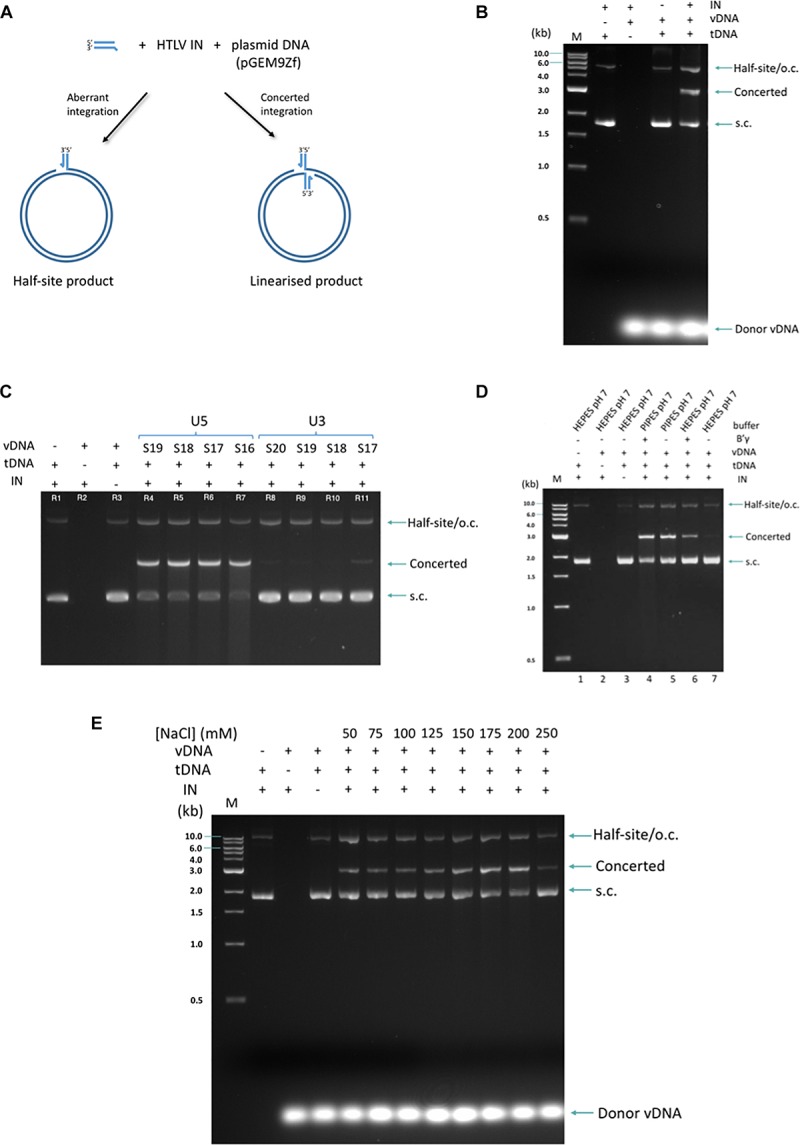
Strand transfer activity of recombinant HTLV-1. **(A)** The formation of two types of product, concerted and half-site, is shown in the schematic. **(B)** The products are resolved on a 1.5% agarose gel and visualized with standard ethidum bromide treatment. M denotes the marker ladder (NEB, 1 kB), vDNA (U5_S18 3′-pre-processed, 18 nucleotide-long, double-stranded oligonucleotide donor mimicking the viral U5 LTR region), tDNA (supercoiled plasmid pGEM9Zf). **(C)** Activity of IN for U5- and U3-derived donors of different lengths is assayed. Sx: substrate of x nt in length. **(D)** HTLV-1 IN is sensitive to the use of different buffering species. When performing the strand transfer reaction in Hepes pH 7, B′γ strongly stimulates the concerted integration activity of HTLV-1 IN. In contrast, under optimal conditions (Pipes pH 7), no B′γ is required for high strand transfer activity. **(E)** Under conditions of optimal activity, IN remains active in the presence of 200 mM NaCl. Migration of DNA species in the gel is indicated on the right of the gel. S.c., supercoiled; o.c., open circular. The 1 kb DNA ladder (NEB, indicated on the left of the gel) was used as a reference. Presence of IN, B′γ, vDNA, tDNA, and buffer conditions are indicated above each gel.

Recombinant IN enzyme was incubated with short (16–20 nt) double-stranded oligonucleotides mimicking the 3′ processed viral LTR end (derived either from the U5 or the U3, vDNA) and a supercoiled plasmid functioning as the target (tDNA) for integration. The biologically relevant concerted integration where two vDNA mimics are inserted in the tDNA, results in linearization of the plasmid, whilst products of aberrant half-site integration co-migrate with nicked plasmid DNA (Figure 1A). These products can be easily separated on an agarose gel (Figure 1B).

Human T-cell lymphotropic virus type 1 IN was active with all donor lengths tested (16–20 nt) but was clearly discriminative against the U3-derived donors (Figure 1C and Supplementary Figure S1). Certain buffering species significantly improved IN *in vitro* strand transfer activity (Figure 1D). PIPES provided such optimal conditions for IN. Indeed, in the PIPES buffer, no B′γ was needed to observe efficient concerted integration activity by HTLV-1 IN. In fact, in this buffer, B′γ only slightly stimulated strand transfer activity of HTLV-1 IN (Figure 1D, compare lanes 4 and 5). Conversely, using the closely related HEPES buffer at the same pH neccesitated the presence of B′γ for any discernable activity (Figure 1D, compare lanes 6 and 7). Finally, in contrast to previous findings where HTLV-1 IN activity was only observed in NaCl concentrations below 100 mM (Muller and Krausslich, 1999), under our optimized conditions, concentrations of up to 200 mM had no adverse effect on activity (Figure 1E).

### Inhibition Profiles of INSTIs in HTLV-1 IN Strand Transfer

**FIGURE 2:**
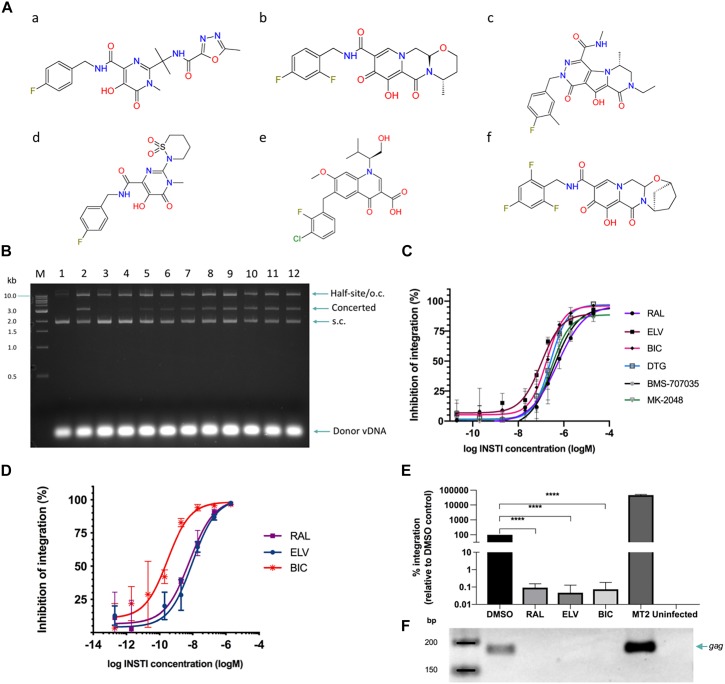
INSTIs efficiently block HTLV-1 integration *in vitro* and HTLV-1 infection by cell-to-cell transmission. **(A)** Chemical structures of the INSTIs used in this study. **(a)** raltegravir, **(b)** dolutegravir, **(c)** MK-2048, **(d)** BMS-707035, **(e)** elvitegravir, and **(f)** bictegravir. Strand transfer is significantly inhibited by the addition of raltegravir **(B)** or other tested INSTIs **(C)**. **(B)** Lane 1, negative control: no IN; lane 2: positive control, IN in the absence of drug. Lanes 3–12: in the presence of raltegravir; lane 3: 200 μM, lane 4: 20 μM, lane 5: 2 μM, lane 6: 634 nM, lane 7: 200 nM, lane 8: 63.4 nM, lane 9: 20 nM, lane 10: 2 nM, lane 11: 200 pM, and lane 12: 20 pM. Migration of DNA species in the gel is indicated on the right of the gel. S.c., supercoiled; o.c., open circular. The 1 kb DNA ladder (NEB, indicated on the left of the gel) was used as a reference. For each of the six compounds tested, the concerted integration bands were quantified by densitometry and values plotted as dose-response curves **(C)**. **(D–F)** INSTIs efficiently block HTLV-1 infection in Jurkat cells. Jurkat cells were pre-treated with INSTIs (2 μM down to 0.2 pM serially diluted 1/10) and infected with HTLV-1 by co-culture with gamma-irrradiated MT-2 cells. Following depletion of MT-2 cells and expansion of the infected Jurkat cells, genomic DNA was isolated. **(D)** Infection was measured by determining the relative PVL of INSTI treated cells compared to DMSO treated control cells. The vertical axis shows the percentage of inhibition of HTLV-1 IN defined as the percentage of (1 – relative PVL). **(E)** Integrated provirus was quantified by Alu-qPCR and normalized to *GAPDH* numbers. DMSO treated Jurkat cells infected with HTLV-1 were arbitrarily set to 100%. Averages and standard deviations from three independent experiments are shown. RAL, raltegravir; ELV, elvitegravir; BIC, bictegravir; Uninfected stands for uninfected Jurkat cells (negative control). Data shown here is for the samples treated with 2 μM of the indicated drug. ^****^: *p*-value = 0.0001 **(F)** Representative gel illustrating products of the nested *gag* PCR (23 cycles) following Alu-PCR. Lanes correspond to the bar graphs in panel **(E)**.

A panel of six INSTIs was selected (Figure 2A), to encompass the inhibitors currently approved for use (raltegravir, elvitegravir, dolutegravir, and bictegravir) as well as those under development (MK-2048, BMS-707035). Strand transfer assays were performed with recombinantly produced IN in presence of each of the respective inhibitors. Although raltegravir and MK-2048 were previously tested to inhibit HTLV-1 (Seegulam and Ratner, 2011), we included them in our study to allow comparison of inhibition profiles under the same assay conditions.

All tested compounds caused significant inhibition of *in vitro* strand transfer at nanomolar concentrations (Figures 2B,C and Table 1). Elvitegravir and the second-generation inhibitors dolutegravir and bictegravir showed activity superior to raltegravir, which is consistent with the observations of these compounds’ activity against HIV-1 IN (Pandey et al., 2007; Marinello et al., 2008; Figure 2C). The biggest improvement was shown by elvitegravir; about 5.3-fold less drug was needed than raltegravir to reach 50% inhibition (Table 1). The Hill slope of the HTLV-1-tested INSTIs oscillates quite consistently around 1.0 – a low Hill slope characteristic of this group of inhibitors (Table 1; Sampah et al., 2011).

**TABLE 1:** Calculated parameters of *in vitro* HTLV-1 IN strand-transfer and HTLV-1 infection inhibition by INSTIs.

	**HTLV-1**						**HIV-1**
	**Inhibition of enzymatic activity**			**Antiviral activity**			**Antiviral activity**
**INSTI**	**IC_50_ (μM)**	**IC_95_ (μM)**	**Hill slope**	**EC_50_ (nM)**	**EC_95_ (μM)**	**CC_50_ (μM)**	**EC_50_ (nM)**
Raltegravir	0.53 ± 0.102	29.92 ± 5.75	0.73	6.42 ± 4.24	1.02 ± 0.675	>20	9.4 ± 1.4^a^
Elvitegravir	0.10 ± 0.016	1.9 ± 0.304	1.0	9.57 ± 5.54	1.27 ± 0.735	>20	2.4 ± 0.9^a^
Dolutegravir	0.26 ± 0.059	3.02 ± 0.485	1.2	ND	ND	ND	1.4 ± 0.3^a^
MK-2048	0.29 ± 0.069	4.22 ± 0.965	1.1	ND	ND	ND	1.9^b^
BMS-707035	0.35 ± 0.074	12.2 ± 2.90	0.83	ND	ND	ND	2.5^c^
Bictegravir	0.18 ± 0.029	2.09 ± 0.437	1.2	0.302 ± 0.173	0.0318 ± 0.0182	>20	1.6 ± 0.4^a^

*^*a*^Tsiang et al. (2016), ^*b*^Bar-Magen et al. (2010), ^*c*^Naidu et al. (2018).*

### Inhibition of HTLV-1 Infection by INSTIs

In order to convincingly establish the usefulness of the selected INSTIs for the purpose of limiting HTLV-1 infection and for potential prophylactic use, we tested a selected panel of INSTIs for their efficacy to block HTLV-1 infection. HTLV-1 is most efficiently spread by cell-to-cell transmission, hence we infected Jurkat cells by co-culture with the persistently HTLV-1 infected MT-2 cell line. To remove any potential carry-over of proviral DNA originating from the MT-2 cells, the MT-2 cells were gamma-irradiated before co-culture with Jurkat cells and depleted using anti-CD25 antibodies conjugated to magnetic beads. Depletion of the MT-2 cells was verified by FACS (Supplementary Figure S2). As an additional control, a condition where only the irradiated MT-2 cells were plated was taken alongside the other conditions. Thus, these cells were used for CD25 depletion and the flow-through was kept in culture and diluted in parallel to the infected Jurkat cells. Following 16 days of expansion, no MT-2 cells were found in these “MT-2 only” conditions further strengthening our observation that the signal observed in our PVL and Alu-PCR assays is specifically derived from newly infected cells.

Infection of Jurkat cells was determined by quantifying the PVL (Demontis et al., 2013). On average 13.4 ± 2.2% (absolute value for the PVL, *n* = 9) of Jurkat cells are infected under these experimental conditions. Infections were done in the presence of DMSO (vehicle of the drugs) or a range of drug concentrations (0.2 pM to 2 μM). The resulting dose-response curves show a strong inhibition profile of tested INSTIs, active even at picomolar concentrations (Figure 2D). This is in-line with numerous observations of INSTIs activity for HIV-1^2^ ^,3^ ^,4^, the INSTI EC_50_ values for both viruses approaching the very low nanomolar range (Table 1). Interestingly, within the context of HTLV-1 replication, bictegravir showed about 20–30-fold higher activity than raltegravir or elvitegravir, respectively. No cellular toxicity was observed at the drug concentrations used (Table 1). Alu-qPCR further confirmed the strong inhibition of integration by these INSTIs (Figures 2E,F). Using this assay, we also investigated the efficacy of tenofovir disproxil fumarate (TDF) to inhibit HTLV-1 transmission. Whilst not as powerful as bictegravir, TDF is still quite potent (EC_50_ = 17.78 ± 7.16 nM), inhibiting HTLV-1 transmission only two- to threefold less efficient than raltegravir or elvitegravir, respectively (Supplementary Figure S3).

## Discussion

Despite the urgent need to curtail HTLV-1 infection and spread, there has been no structural information available on the delta-retroviral IN, and the data on its inhibition by the HIV-1 INSTIs is outdated. By successful recombinant expression, purification and further characterization of activity of full-length HTLV-1 IN, we have laid the groundwork for accurate assessment of its inhibition by a panel of selected INSTIs. In contrast to a previous study which first described the *in vitro* 3′-processing, strand transfer and disintegration activity of HTLV-1 IN using radiolabeled probes (Muller and Krausslich, 1999), the advantage of the integration assay employed here is that the biologically relevant concerted integration activity can be discerned from the aberrant half-site integration events and quantified separately. Stabilization of IN at high concentrations also allows to avoid the use of radioactivity. By optimizing conditions for HTLV-1 IN activity, the need for the presence of detergent and the host factor B′γ for efficient strand transfer activity has been removed. Furthermore, the observation of equal stimulation by either B′γ or optimized buffering conditions suggests that the said *in vitro* stimulation of HTLV-1 IN activity by B′γ occurs by its entropic stabilization upon binding of the enzyme, rather than by a specific mechanism improving substrate recognition or catalysis. It has also been shown that HTLV-1 IN is active on a range of vDNA LTR donor mimicks with lengths as short as 16 bp – although, as reported previously (Muller and Krausslich, 1999), it strongly discriminates against the U3-derived donors. The discrimination between U5 and U3 ends is not unique for HTLV-1 IN, indeed HIV-1 and SIV IN also display a similar preference for U5 LTR ends (Cherepanov et al., 1999; Goodarzi et al., 1999). Although the presence of half-site product is apparent, under optimized conditions the concerted integration product predominates (Figure 1).

The similarity of HTLV-1 IN INSTI sensitivity to that of HIV-1 is striking (Table 1; Tsiang et al., 2016). The apparent disparity between the *in vitro*- and *in cellulo*-derived IC_50_ values is caused by the stark difference in IN concentration to be inhibited in these assays. Since IN is known not to form a steady-state or turn over (Dicker et al., 2007), its concentration is positively correlated with the resulting INSTI IC_50_. Furthermore, in our cell-to-cell infection model, bictegravir performed about 20- to 30-fold better than either raltegravir or elvitegravir. In *in vitro* assays, bictegravir is only three times as active as raltegravir, and ∼55% as active as elvitegravir. Why bictegravir is so much more efficient in blocking HTLV-1 infection than *in vitro* integration remains enigmatic. We speculate that Jurkat cells used in our experiments might be more efficient in taking up bictegravir than raltegravir or elvitegravir, compared to the MT-2 or primary T cells used by Tsiang et al. (2016).

Recently, Pasquier et al. (2018) reported a thorough study investigating the efficacy of HIV-1 reverse transcriptase (RT) inhibitors in blocking HTLV-1 infection. Whilst zidovudine was able to block HTLV-1 infection somewhat (<10–20% inhibition at 50 μM concentration), lamivudine and stavudine were not (Pasquier et al., 2018). Tenofovir disproxil fumarate were shown to inhibit HTLV-1 transmission; at concentrations of 10 μM about 50% of HTLV-1 infection was blocked (Pasquier et al., 2018). In our hands, we measured an EC_50_ of 17.78 ± 7.16 nM for TDF. The discrepancy is likely due to the different assays used. Whilst Pasquier et al. (2018), reads out luciferase activity from an HTLV-1 LTR promoter 24 h post co-culture with live C91PL cells, we deplete the gamma-irradiated MT-2 cells and measure (integrated) proviral DNA 16 days post-infection. Nevertheless, this is very promising data. Indeed, whilst zidovudine in combination with IFN-alpha or valproic acid was shown to reduce PVL in some cases (Afonso et al., 2010; Trevino et al., 2012; Cook et al., 2018), there is hope for improvement. The fact that INSTIs are as potent as TDF, and in the case of bictegravir ∼60 more potent than TDF in blocking HTLV-1 infection (EC_50_ values between 0.3 and 10 nM, Figures 2D–F), may justify the inclusion of INSTIs in clinical trials.

The occurrence of escape mutants is a real challenge in HIV-1 patients and drives the research toward the design of alternative anti-retroviral therapy (Harada and Yoshimura, 2017). The mutation rate of HTLV-1 RT is about one fourth of HIV-1 RT (Mansky, 2000). This alone cannot explain the apparent lack of HTLV-1 quasi species, which is in part due to its different mode of replication. Although limited clinical data is available, it is promising that so far, no resistance mutations have been observed in HTLV patients experiencing a strong reduction in PVL upon treatment with RT inhibitors for more than 1 year (Machuca et al., 2001).

**FIGURE 3:**
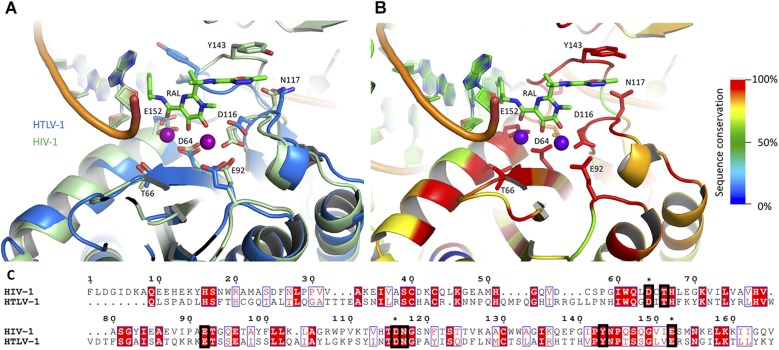
The active sites of HIV-1 and HTLV-1 IN are highly conserved. A homology model of HTLV-1 CCD is presented **(A)**, overlaid on a previously reported model of HIV-1 intasome, based on the crystal structure of PFV intasome with raltegravir present in the active site (Krishnan et al., 2010). **(C)** A pairwise sequence similarity between the HIV-1 and HTLV-1 INs was conducted with MAFFT (Katoh and Standley, 2013), and the resulting similarity score for each residue was overlaid on the structure, represented in a color scale (red for highest conservation through to blue for lowest/no conservation), using the Alebrije script **(B)**. The catalytic DDE triad as well as residues known to be involved in INSTI resistance are shown in stick representation **(B)** and indicated in the alignment with black squares **(C)**. The residues of the DDE motif are also marked with asterisks. Catalytically important magnesium atoms are represented in purple **(B)**. The predominance of residues highly conserved between the two INs is apparent within the active site. Pairwise alignment represented using ESPRIPT (Gouet et al., 1999), the model in panel **(B)** was produced using PyMOL (Schrodinger, 2015).

Taken together, our results strongly suggest that HTLV-1 IN is as sensitive to the tested panel of INSTIs as HIV-1. Although there are significant differences in the overall sequence similarity between the two proteins as well as the infection dynamics of these viruses, the residues within the active site are highly conserved (Figure 3). Whilst it is tempting to speculate why elvitegravir is fivefold better in inhibiting *in vitro* HTLV-1 IN activity, we note that the loop important for drug interaction (including the homologous residues to HIV-1 Y143, N144, and Q146) is not well structured in our HTLV-1 homology model (Figure 3A). It is therefore difficult, at this stage, to explain the differences in inhibition efficiency between raltegravir and elvitegravir. A structure of the HTLV-1 (or related delta-retroviral) intasome bound to INSTIs is needed. In addition, structural information available on bictegravir bound to a retro- or lentiviral intasome is currently lacking.

Early on in infection, HTLV-1 proliferates mostly by viral replication. Unlike HIV-1, these dynamics change dramatically a few weeks later, where the PVL is most significantly increased by the expansion of infected clones and viral replication is turned down. The time window to use anti-retrovirals in recently HTLV-1 exposed patients to block infection is very narrow (Cook et al., 2016). It is well known that a high PVL increases the odds of developing HAM/TSP (Nagai et al., 1998; Matsuzaki et al., 2001; Takenouchi et al., 2003; Olindo et al., 2005, 2006) and ATLL (Iwanaga et al., 2010; Yonekura et al., 2015). Therefore, reducing the PVL by use of antiretrovirals might prevent an asymptomatic carrier from developing HTLV-1 associated disease. HTLV-1 screening of organ donors is not standard procedure. Recent analysis of living organ donor recipients that were HTLV-1 negative before transplantation show a high incidence of seroconversion and development of HAM (40%) within 4 years of transplantation (Taylor, 2018; Yamauchi et al., 2019). Whether the fast development of HAM is correlated with immunosuppressive therapy is unclear but use of potent INSTIs (either given to the donor to reduce PVL in the organ at the time of explant or administered peri-transplantation) could possibly reduce transmission rate. A neat study in which naturally STLV-1 infected baboons were treated with a combination of valproic acid and zidovudine showed a decline in PVL (Afonso et al., 2010); Cook et al. (2018) reported the long-term clinical remission after cessation of zidovudine in combination with interferon-alpha in one patient diagnosed with chronic ATL; and Soriano and colleagues on the other hand reported a decrease in PVL in raltegravir treated HAM patients but not in asymptomatic carriers (Trevino et al., 2012).

The presented data should encourage the use of INSTIs and bictegravir in particular, especially in the preventative treatment for HTLV-1 transmission among drug users, serodiscordant couples, organ recipients and pregnant/breastfeeding mothers. Moreover, more clinical trials are needed to investigate the efficacy of using anti-retrovirals (combining RT and INSTI inhibitors with or without IFN-alpha or valproic acid) in reducing PVL thereby reducing chances of transmission between partners, but also in cases of organ donor transplantation, and possibly preventing the evolution of asymptomatic carrier to HTLV-1 associated disease. Treating pregnant women with INSTIs might warrant some caution; given the reduced exposure of elvitegravir during pregnancy it increases the risk of virological failure and mother-to-child transmission (Momper et al., 2018; van der Galien et al., 2019). The WHO issued a drug safety alert for dolutegravir^5^ following the observation that children born to HIV-1 positive mothers treated with dolutegravir at the time of conception were more likely to have neural tube defects (Zash et al., 2018). Data obtained from a recent study in France did not support a pharmacovigilance signal on neural tube defects in women exposed to dolutegravir, raltegravir or elvitegravir (Chouchana et al., 2019). The limitation of all these studies are the small number of patients involved. Nevertheless, caution is warranted and careful monitoring and sharing of information in regard to anti-retroviral therapy during pregnancy to investigate the risks involved, is paramount. Bictegravir has only recently been FDA approved^6^, thus investigations are needed to see whether this INSTI, a close relative of dolutegravir, is safe to use during pregnancy. For now, raltegravir is the recommended INSTI to suppress viral load in HIV-1 pregnant women and prevent mother-to-child transmission, and no adverse effects have been found (Chouchana et al., 2019; Rasi et al., 2019; van der Galien et al., 2019).

Currently, the most devastating pathology caused by HTLV-1 is ATLL. Prognosis is very poor – most patients die within several months after presentation (Beltran et al., 2011). At the same time, it is known that the likelihood of an HTLV-1 carrier developing ATLL is correlated with exposure early in life – most commonly during breastfeeding or child-birth (Carneiro-Proietti et al., 2014). In conclusion, using INSTIs as a prophylactic could have a dramatic impact on limiting the spread of the virus within many high-risk populations and preventing the development of ATLL among them.

## Data Availability

All datasets generated for this study are included in the manuscript and/or the Supplementary Files.

## Author Contributions

MB designed, executed, and analyzed the experiments, and wrote the manuscript. JM designed, executed, and analyzed the experiments, and edited the manuscript. GM designed and analyzed the experiments, and wrote the manuscript.

## Conflict of Interest Statement

The authors declare that the research was conducted in the absence of any commercial or financial relationships that could be construed as a potential conflict of interest.

## Footnotes

1 https://github.com/mbarski/Alebrijes

2 https://www.accessdata.fda.gov/drugsatfda_docs/label/2013/022145s029lbl.pdf (accessed December 20, 2018)

3 https://www.accessdata.fda.gov/drugsatfda_docs/label/2014/203093s000lbl.pdf (accessed December 20, 2018)

4 https://www.accessdata.fda.gov/drugsatfda_docs/label/2013/204790lbl.pdf (accessed December 20, 2018)

5 https://www.who.int/medicines/publications/drugalerts/Statement_on_DTG_18May_2018final.pdf?ua=1 (accessed May 07, 2019)

6 https://www.accessdata.fda.gov/drugsatfda_docs/label/2018/210251s000lbl.pdf (accessed December 20, 2018)
