# Supplementary material for: Inhibition of HTLV-1 Infection by HIV-1 First- and Second-Generation Integrase Strand Transfer Inhibitors
Source: Front Microbiol. 2019 Aug 13;10:1877. doi: 10.3389/fmicb.2019.01877 (PMC6705210; doi:10.3389/fmicb.2019.01877)
Supplement: Supplementary file 1 [file Data_Sheet_1.docx]

**SUPPLEMENTARY INFORMATION**

**Supplementary Table S1.** List of primers used for strand-transfer assays as mimics of the U5 and U3 LTR regions.

| **Name** | **Sequence** |
| --- | --- |
| U5_S16UP | AAATTTAGTACACA |
| U5_S16B | ACTGTGTACTAAATTT |
| U5_S17UP | GAAATTTAGTACACA |
| U5_S17B | ACTGTGTACTAAATTTC |
| U5_S18UP | AGAAATTTAGTACACA |
| U5_S18B | ACTGTGTACTAAATTTCT |
| U5_S19UP | GAGAAATTTAGTACACA |
| U5_S19B | ACTGTGTACTAAATTTCTC |
| U5_20UP | AGAGAAATTTAGTACACA |
| U5_S20B | ACTGTGTACTAAATTTCTCT |
| U5_S20UPQ | GACTCACTATAGGGCACGCGTAGAGAAATTTAGTACACA |
| U5_S20BQ | ACTGTGTACTAAATTTCTCTACGCGTGCCCTATAGTGAGTC |
| U3_S17UP | TCATGGTCATTGTCA |
| U3_S17B | GATGACAATGACCATGA |
| U3_S18UP | CTCATGGTCATTGTCA |
| U3_S18B | GATGACAATGACCATGAG |
| U3_S19UP | GCTCATGGTCATTGTCA |
| U3_S19B | GATGACAATGACCATGAGC |

**Supplementary Table S2.** Primers used for Proviral Load determination by qPCR

| **Name** | **Sequence** |
| --- | --- |
| SK43 | CGGATACCCAGTCTACGTGT |
| SK44 | GAGCCGATAACGCGTCCATCG |
| GAPDH-Fwd | AACAGCGACACCCATCCTC |
| GAPDH-Rev | CATACCAGGAAATGAGCTTGACAA |
| Alu-Fwd | CCTCCCAAAGTGCTGGGATTACA |
| Gag-Rev | GGCTTGGGTTTGGATGAGTA |
| Gag-Fwd | CCCTCCAGTTACGATTTCCA |

**SUPPLEMENTARY FIGURES**

**
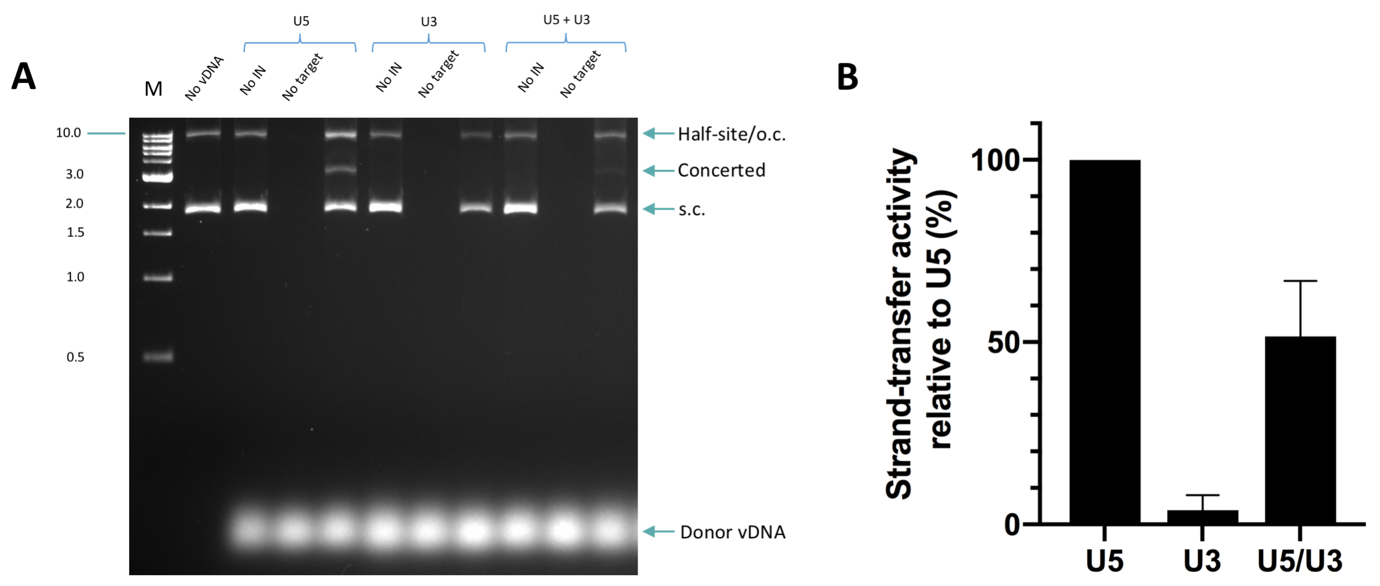
**

**Supplementary Figure S1.** HTLV-1 IN strand-transfer reactions were performed with vDNA donor derived from the U5 or U3 regions, or an equimolar mixture of both U5 and U3-mimicking donors. Discrimination against the U3 donor by the HTLV-1 IN *in vitro* is apparent, both alone as well as when mixed with its U5 counterpart **(A)**. Densitometry quantification of this experiment, performed in triplicate, is also shown **(B)**.

**Supplementary Figure S2.** MT-2 cells were successfully depleted from the co-culture as indicated by FACS analysis. Samples were stained with anti-CD3-Alexa647 and anti-CD25-Alexa488 antibodies to allow distinction between the two cells types (Jurkat cells are CD3^+^ and MT2 are CD25^+^). Panel (A) Jurkat cells alone, (B) MT-2 alone, (C) Jurkat cells mixed with MT-2, (D) Jurkat cells + MT-2 following MT-2 depletion, (E) histogram of CD25^+^ cells and (F) histogram of CD3^+^ cells used for the quantification of Jurkat and MT-2 cells in the sample. (D) table indicating the % of CD3^+^ (Jurkat) and CD25^+^ MT-2 cells (derived from panels E and F).

EC_50_=17.78 ± 7.16 nM

**Supplementary Figure S3.** Tenofovir disproxil fumarate (TDF) inhibits HTLV-1 transmission. Jurkat cells were treated with TDF (20 μM to 20 nM in serial 10-fold dilutions) before infection by co-culture with gamma-irradiated MT-2 cells as described in Materials and Methods. Following depletion of MT-2 cells and expansion of the cells, genomic DNA was extracted and the PVL was determined. Data shown are relative to DMSO control, averages and standard deviations are from three biological replicates.
